# Modelling associations between neurocognition and functional course in young people with emerging mental disorders: a longitudinal cohort study

**DOI:** 10.1038/s41398-020-0726-9

**Published:** 2020-01-21

**Authors:** Jacob J. Crouse, Kate M. Chitty, Frank Iorfino, Joanne S. Carpenter, Django White, Alissa Nichles, Natalia Zmicerevska, Adam J. Guastella, Elizabeth M. Scott, Rico S. C. Lee, Sharon L. Naismith, Jan Scott, Daniel F. Hermens, Ian B. Hickie

**Affiliations:** 1 Youth Mental Health Team, Brain and Mind Centre, The University of Sydney, Camperdown, NSW Australia; 2 The University of Sydney Faculty of Medicine and Health, Discipline of Pharmacology, Translational Australian Clinical Toxicology Group, Sydney, NSW Australia; 3 InnoWell, Pty Ltd., Sydney, NSW Australia; 4 The University of Notre Dame, St Vincent’s and Mater Clinical School, Sydney, NSW Australia; 5 Turner Institute for Brain and Mental Health, Monash University, Clayton, VIC Australia; 6 Healthy Brain Ageing Program, Brain and Mind Centre, The University of Sydney, Camperdown, NSW Australia; 7 Charles Perkins Centre, The University of Sydney, Camperdown, NSW Australia; 8 Academic Psychiatry, Institute of Neuroscience, Newcastle University, Newcastle, UK; 9 Sunshine Coast Mind and Neuroscience Thompson Institute, University of the Sunshine Coast, Birtinya, QLD Australia

**Keywords:** Bipolar disorder, Schizophrenia, Depression

## Abstract

Neurocognitive impairment is commonly associated with functional disability in established depressive, bipolar and psychotic disorders. However, little is known about the longer-term functional implications of these impairments in early phase transdiagnostic cohorts. We aimed to examine associations between neurocognition and functioning at baseline and over time. We used mixed effects models to investigate associations between neurocognitive test scores and longitudinal social and occupational functioning (“Social and Occupational Functioning Assessment Scale”) at 1–7 timepoints over five-years in 767 individuals accessing youth mental health services. Analyses were adjusted for age, sex, premorbid IQ, and symptom severity. Lower baseline functioning was associated with male sex (coefficient −3.78, 95% CI −5.22 to −2.34 *p* < 0.001), poorer verbal memory (coefficient 0.90, 95% CI 0.42 to 1.38, *p* < 0.001), more severe depressive (coefficient −0.28, 95% CI −0.41 to −0.15, *p* < 0.001), negative (coefficient −0.49, 95% CI −0.74 to −0.25, *p* < 0.001), and positive symptoms (coefficient −0.25, 95% CI −0.41 to −0.09, *p* = 0.002) and lower premorbid IQ (coefficient 0.13, 95% CI 0.07 to 0.19, *p* < 0.001). The rate of change in functioning over time varied among patients depending on their sex (male; coefficient 0.73, 95% CI 0.49 to 0.98, *p* < 0.001) and baseline level of cognitive flexibility (coefficient 0.14, 95% CI 0.06 to 0.22, *p* < 0.001), such that patients with the lowest scores had the least improvement in functioning. Impaired cognitive flexibility is common and may represent a meaningful and transdiagnostic target for cognitive remediation in youth mental health settings. Future studies should pilot cognitive remediation targeting cognitive flexibility while monitoring changes in functioning.

## Introduction

Reducing the burden of disability attributable to mental disorders is a global health priority. Mental, neurological, and substance use disorders are the world’s leading contributors to years lived with disability and the third-ranked cause of disability-adjusted life years^1,2^. This disability burden is particularly heavy for young people. For example, disability-adjusted life years related to common mental disorders reach their peak between ages 10–29 years^2^, and depression, bipolar disorder, and schizophrenia are three of the four most burdensome conditions in those aged 10–24 years^3^. As 50% of mental disorders emerge before the middle-teens and 75% by the mid-twenties^4^, it is likely that the burden of disability in early and later adulthood represents an extension of problems originating premorbidly or in early phases of illness. These early phases are characterized by changes in social behaviors and difficulties participating in work and study^5^, both of which are reflected in the high rates of functional impairment at presentation to youth-specific services^6,7^. As functional trajectories are heterogeneous and often persistently impaired^6^, there is a critical need for identification of factors associated with functional course which may guide interventions to those at risk of enduring and lifelong functional disability^8^.

Despite clinical remission, many individuals with mental disorders fail to reach their expected or premorbid levels of social and occupational functioning, suggesting the presence of enduring factors which limit functional recovery. One such factor is neurocognitive impairment, which is common from early in the course of depressive^9^, bipolar^10^, and psychotic disorders^10,11^. While there is good evidence to suggest that neurocognitive impairments represent an enduring and trait-like feature of neurodevelopmental disorders such as schizophrenia^12^, the state-trait distinction is less clear for other mental disorders. However, some evidence suggests that attentional and other executive impairments commonly persist despite remission of symptoms in major depression^9,13^, while domains such as memory, verbal fluency, and processing speed are more strongly influenced by mood state^9,13^. Likewise, impairments in memory, processing speed, and executive functions are common in individuals with bipolar disorder across mood episodes^14,15^, notwithstanding common inter-episode syndromal and sub-syndromal symptoms ^16^.

The functional consequences of neurocognitive impairments in people with mental disorders are increasingly clear. Research over the last two decades has demonstrated that neurocognition is a strong and prospective determinant of functioning in schizophrenia^17^, with impairments in domains including processing speed, executive functions, and memory^18^ limiting patients’ capacity to acquire, retain, and relearn skills required for adaptive functioning^17^. More recently, similar impacts have been appreciated for a wider range of mental disorders. Longitudinal studies of people with bipolar disorder have demonstrated associations between impairments in executive functions^14,19^, processing speed^14,20^, and verbal learning and memory^14,19,20^ and poorer social and occupational outcome. While a less developed literature, poorer memory and executive functions have also been linked to worse follow-up social and occupational outcome in major depression^21,22^.

Importantly, there is a developing notion that neurocognition may represent a continuum cutting across diagnostic boundaries^10,23^, with the National Institute of Mental Health’s Research Domain Criteria endorsing a dimensional framework to the study of neurocognition in mental disorders^24^. Our group has demonstrated the utility of a transdiagnostic approach to examining neurocognition and functioning across the major mental disorders. We have reported strong cross-sectional^25^ and longitudinal^23,26^ relationships between general neurocognition and social and occupational functioning in a cohort of young people presenting to mental health services with a range of mood, anxiety, and psychotic syndromes. Moreover, we have shown that changes in neurocognition map onto changes in functioning when statistically adjusting for diagnosis and symptom severity, supporting a meaningful and robust link between neurocognition and functioning across mental disorders^27^. Limited work however has examined relationships between specific neurocognitive domains and the course of functioning in young transdiagnostic samples.

Accordingly, we aimed to test several questions regarding the links between neurocognition and functioning and their broader implications across mental disorders in a cohort of adolescents and young adults accessing mental health services. First, we aimed to examine associations between neurocognitive test scores across nine domains and functioning at baseline and change in functioning over time. Second, we aimed to determine whether associations between neurocognition and functioning at baseline and change in functioning over time would be robust to adjustment for confounding factors (age, sex, premorbid IQ, and symptom severity). Based on our work^23,26^ and the wider literature^19,20,28^, we hypothesized that baseline executive functions, processing speed, and verbal learning and memory would be uniquely associated with baseline functioning and change in functioning longitudinally.

## Materials and methods

### Human ethics

The study and consent procedure were approved by the University of Sydney Human Research Ethics Committee (project numbers 2012/1626 and 2012/1631) and conducted in accordance with the revised Declaration of Helsinki. All participants aged 16 and older provided written informed consent and parental or guardian consent was obtained for participants aged under 16 years.

### Participants

Participants were drawn from a cohort of 6743 consecutive referrals (aged 12–30) presenting to youth mental health clinics at the Brain and Mind Center in Sydney, Australia, who were recruited to a research register of adolescents and young adults with mental disorders between 2008–2018. These clinics (e.g., *headspace*) aim to provide youth-friendly and highly accessible early intervention services for young people with emerging mental and substance use disorders^29^. *Headspace* consists of an integrated mix of primary-level services and more specialized services (e.g., drug and alcohol) and primarily attracts young people with a wide range of mental health problems (typically anxiety, mood and/or psychotic syndromes). All participants were receiving ongoing clinician-based case management and relevant social, psychological and/or medical treatments as part of standard care, which may have involved contact with a psychiatrist, psychologist, occupational therapist, support worker or hospitalization for those whose need exceeded the capacity of the primary care services.

### Eligibility criteria

Inclusion criteria for this study were: (a) a baseline neurocognitive assessment with the majority of test scores available; (b) aged 12–30 at the neurocognitive assessment; (c) an available proforma assessment (see below) within three-months of the neurocognitive assessment; and (d) willing and able to provide written informed consent (or parental/guardian consent was obtained). Exclusion criteria were: (i) history of neurological disease; (ii) medical illness known to impact brain function (e.g., cancer, epilepsy); (iii) electroconvulsive therapy in three-months prior to neurocognitive assessment; (iv) clinically-evident intellectual disability; and/or (v) insufficient understanding of the English language to allow participation in verbal assessments or testing.

### Data collection (baseline)

A subset of the wider cohort participated in detailed clinical and neurocognitive assessments between 2008–2015. A board-certified neuropsychologist, research psychologist or supervised doctoral student administered a neurocognitive battery with the domains chosen on the basis of sound validity and reliability^30^, relevance to the diagnoses under study^9,11,31^, and overlap with instruments used in the Measurement and Treatment Research to Improve Cognition in Schizophrenia (MATRICS) initiative^32^. The following domains were assessed: *processing speed* (Trail Making Test, part A)^33^, *cognitive flexibility* (Trail Making Test, part B)^33^, *verbal learning* (sum of trials 1–5 of the Rey Auditory Verbal Learning Test; RAVLT)^34^, *verbal memory* (20-min delayed recall of the RAVLT)^34^, *sustained attention* (A’ Prime subtest of the Rapid Visual Information Processing Test)^35^, *set-shifting* (Intra-Extra Dimensional Set Shift)^35^, *visuospatial learning* (Paired Associates Learning Task)^35^ and *working memory* (Spatial Span Task)^35^. Premorbid IQ was estimated using the Wechsler Test of Adult Reading^36^ or the Wide Range Achievement Test^37^ (for participants younger than 16). Neurocognitive scores were standardized to age-matched and sex-matched norms (*z*-scores) using established norms^38,39^. To limit the impact of extreme scores and minimize data transformation, *z*-scores were curtailed at a maximum of ±5.0, with fewer than 3% of scores curtailed for each test. Symptom type and severity were determined using the Brief Psychiatric Rating Scale, with four dimensions empirically derived (depressive, negative, positive, and manic)^40^.

### Data collection (longitudinal)

A standardized clinical proforma was used to retrospectively extract demographic, clinical, and functioning data from clinical and research files across eight predetermined timepoints (baseline, 3 months, 6 months, 1 year, 2 years, 3 years, 4 years, and 5 years)^41^. A “time-last-seen” entry was also recorded; however, this was not included in the current study. The proforma captures information at each timepoint regarding the current presentation and illness course, including: (a) demographics; (b) socio-occupational functioning; (c) clinical presentation (including clinical diagnosis according to DSM-5^42^); (d) self-harm and suicidal thoughts and behaviors; (e) alcohol and other substance use; and (f) physical health comorbidities.

The proforma provided the primary outcome measure of socio-occupational functioning as assessed by a trained clinician using the Social and Occupational Functioning Assessment Scale (SOFAS). The SOFAS is a 100-point scale (with higher scores denoting better functioning) which improves on other measures of global functioning in its instruction to the rater to avoid confounding the rating with symptoms. A score of 60–70 is indicative of moderate difficulty in social, occupational, or school functioning. The SOFAS is widely used and has good construct validity^43^, inter-rater reliability^43^, and predictive validity ^44^.

As neurocognition was the primary baseline predictor for this study, we used the nearest proforma assessment occurring within a three-month interval of the neurocognitive assessment as the participants’ baseline timepoint (T1), with remaining proforma timepoints recoded if necessary. As we allowed a three-month interval for recoding, we subsequently excluded the three-month proforma timepoint from further analysis. The number of proforma assessments at each timepoint and the number of participants with one or more proforma assessments over time are presented in Supplementary Tables 1, 2, respectively.

### Statistical analyses

Analyses were conducted in RStudio (version 1.0.143). Linear mixed-effects models with random-intercepts were constructed using the “lme4” package (version 1.1–18–1^45^). Full-information maximum-likelihood estimation was used to handle missing follow-up data (as loss to follow-up was uncontrolled). The mixed-effects framework is recommended for longitudinal designs as it tolerates: (a) repeated-measures within participants (i.e., non-independence); (b) unbalanced assessment intervals; and (c) missing follow-up data. The continuous SOFAS rating at each timepoint represented the outcome variable, and participants could contribute one or multiple assessments over time (i.e., assessments nested within participants) (see Supplementary Table 2). To model associations with the rate of change in SOFAS over time, a “Time” variable was used which represented the timepoint of each assessment and was linearly coded. All baseline predictor variables were continuous (except for sex).

The literature describing relationships between neurocognitive test performance and aspects of functioning in major mental disorders report associations between a large variety of neurocognitive tests and various measures of global functioning and specific subdomains of functioning (e.g., relationship impairment, work impairment, and independent living)^14,18–22^. Relatedly, most studies have focussed on specific diagnostic groups (e.g., schizophrenia) and there is very limited research regarding specific neurocognition-functioning associations in early-phase, transdiagnostic cohorts. Accordingly, we chose to use a data-driven, backward elimination statistical approach to identify associations between individual neurocognitive test scores and social and occupational functioning in our cohort. Modeling proceeded in three stages. First, we examined unadjusted associations between all baseline predictors and variation in SOFAS scores at baseline as well as the rate of change in SOFAS over time. Second, we examined associations between all baseline predictors and variation in baseline SOFAS scores, using backward elimination to iteratively remove the least significant variable until only significant predictors remained (*α* = 0.05). Third, we examined associations between the rate of change in SOFAS longitudinally and all predictor variables that had significant associations with variation in SOFAS at baseline, using backward elimination to reduce the full model.

Normality of residuals was inspected with Q–Q plots, with an approximate normal distribution evident. Multicollinearity was assessed with the variation inflation factor (VIF), with no predictors exceeding a VIF of 3.0. Parameter-specific *p*-values were calculated using Satterthwaite’s approximation for degrees of freedom in the “lmerTest” package (version 1.0^46^). Missing baseline neurocognitive and clinical data were imputed using multiple imputation by chained equations in the “mice” package (version 3.3.0^47^). Missing data patterns were consistent with a missing-at-random mechanism and fewer than 10% of each neurocognitive domain and fewer than 12% of BPRS scores were missing (see Supplementary Table 3 for numbers and proportions of missing values for each predictor variable). Following recommendations, we multiply imputed 100 datasets using predictive mean matching (which makes use of all available data), modeled each imputed dataset separately, and pooled the coefficients, test statistics, and *p*-values^47–49^.

### Role of the funding source

This study was partially funded by an Australian Government Research Training Program Scholarship (awarded to J.J.C.), a National Health & Medical Research Council Center of Research Excellence Grant (No. 1061043) and an Australia Fellowship (No. 511921) (awarded to I.B.H.). The funders of this study had no involvement in the: study design; collection, analysis and reporting of the data; writing of the report; or decision to submit the paper for publication.

## Results

### Sample characteristics

Participants were drawn from a cohort of 6743 young people who were recruited to a research register^50^. Of these, 2767 participants had an available baseline clinical proforma assessment, and a total of 767 participants met all eligibility criteria and were included in the final analysis.

Sample characteristics are presented in Table 1. At baseline, the sample consisted of 767 participants (409/767 female; 53.3%) with a median age of 19 years (IQR = 6). Baseline SOFAS ratings ranged from 30 to 90 with a mean in the moderate-impairment range (mean [SD] 60.19 [10.05]) (mean SOFAS scores at each timepoint are reported in Supplementary Table 4). Numbers and proportions of participants with SOFAS outcome data at follow-up timepoints were: 6 months (*N* = 247, 32.2%); 1 year (*N* = 275, 35.5%); 2 years (*N* = 236, 30.8%); 3 years (*N* = 170, 22.2%); 4 years (*N* = 112, 14.6%); and 5 years (*N* = 59, 7.7%). Around two-thirds of the sample had two or more timepoints (*N* = 465, 60.6%) and over one-third had three or more timepoints (*N* = 36.9%) (see Supplementary Table 2).

**Table 1:** Baseline socio-demographics, clinical symptom ratings and neurocognitive scores in 767 help-seeking young people (aged 12–30 years at baseline) accessing mental health services.

	*N* (%) or Mean ± SD
Socio-demographics	
Sex (female)	409 (53.3)
Age (years)	19.82 ± 4.11
SOFAS	60.19 ± 10.05
Premorbid IQ	102.19 ± 10.11
Clinical symptom ratings^a^	
BPRS depressive	13.5 ± 5.23
BPRS negative	7.30 ± 2.91
BPRS positive	10.70 ± 3.77
BPRS manic	9.40 ± 3.16
Neurocognition^b^	
Processing speed (TMT-A)	−0.07 ± 1.14
Cognitive flexibility (TMT-B)	−0.65 ± 1.56
Verbal learning (RAVLT-sum)	−0.31 ± 1.33
Sustained attention (RVP-A)	−0.70 ± 1.35
Verbal memory (RAVLT-A7)	−0.30 ± 1.35
Verbal fluency (COWAT)	−0.35 ± 1.12
Working memory (SSP)	0.00 ± 1.14
Visuospatial learning (PAL)	−0.26 ± 1.32
Set shifting (IED)	−0.41 ± 1.46

*SOFAS* Social and Occupational Functioning Assessment Scale, *Premorbid IQ* estimated premorbid intellectual functioning, *BPRS* Brief Psychiatric Rating Scale, *TMT-A* Trail Making Test part A, *TMT-B* Trail Making Test part B, *RAVLT-sum* Rey Auditory Verbal Learning Test sum of trials 1–5, *RVP* Rapid Visual Processing Task, A-Prime subtask, RAVLT-A7 = 20-minute delayed recall, *COWAT* Controlled Oral Word Association Test, *SSP* Spatial Span Task, *PAL* Paired Associates Learning, *IED* Intra-extra Dimensional set shift

^a^Mean and SD based on non-imputed data with missing values (fewer than 12%)

^b^Mean and SD based on non-imputed data with missing values (fewer than 10%).

Presenting primary mental health diagnoses are shown in Table 2. The majority of patients presented with a primary mood (depressive or bipolar) or anxiety disorder (*N* = 523/767, 68.2%). Level of symptoms on the BPRS were in the “very mild” to “mild” range across the four dimensions (depressive, negative, positive, and manic). The means for each neurocognitive domain were within normal limits. Only scores for cognitive flexibility (mean [SD] −0.65 [1.56]) and sustained attention (mean [SD] −0.70 [1.35]) exceeded −0.5 SD below the norm, while all other domains fell within 0 and −0.5 SD of the norm: processing speed (mean [SD] −0.07 [1.14]), verbal learning (mean [SD] −0.31 [1.33]), verbal memory (mean [SD] −0.30 [1.35]), verbal fluency (mean [SD] −0.35 [1.12]), visuospatial learning (mean [SD] −0.26 [1.32]), set-shifting (mean [SD] −0.41 [1.46]) and working memory (mean [SD] 0.00 [1.14]). Clinically significant impairment (i.e., −1.5 SD or greater below the norm) was common across all domains: working memory (8.6%), processing speed (10.2%), visuospatial learning (11.5%), verbal fluency (14.3%), set-shifting (16.5%), verbal learning (16.9%), verbal memory (20.6%), cognitive flexibility (23.0%), and sustained attention (27.4%).

**Table 2:** Presenting primary diagnoses of 767 help-seeking young people (aged 12–30 at baseline) accessing mental health services.

Presenting primary diagnosis	*N* (%)
Depressive disorders	292 (38.1)
Bipolar and related disorders	102 (13.3)
Schizophrenia spectrum and other psychotic disorders	98 (12.8)
Anxiety disorders	129 (16.8)
Obsessive-compulsive and related disorders	11 (1.4)
Trauma-related and stressor-related disorders	14 (1.8)
Neurodevelopmental disorders	46 (6.0)
Feeding and eating disorders	4 (0.5)
Personality disorders	7 (0.9)
Disruptive, impulse-control, and conduct disorders	23 (3.0)
Substance use and addictive disorders	13 (1.7)
No diagnosis/uncertain diagnosis	28 (3.7)

### Unadjusted associations with baseline functioning and change in functioning over time

We first modeled associations between all baseline predictors and variation in SOFAS at baseline and variation in the rate of SOFAS change longitudinally. As presented in Supplementary Table 5, all variables (except for age at baseline) in the unadjusted models were significantly associated with baseline functioning. Significant positive associations with baseline functioning were observed for all nine neurocognitive domains and premorbid IQ, while significant negative associations were observed for male sex and depressive, negative, positive, and manic symptoms. There were significant and positive associations with the rate of SOFAS change longitudinally for cognitive flexibility, verbal learning, verbal memory, working memory, processing speed, male sex, baseline age, and depressive, positive, and manic symptoms.

### Associations with baseline functioning and change in functioning over time, adjusted for socio-demographics and type and severity of symptoms

We next used backward elimination to reduce the full model including all predictor variables down to a final model in which only variables significantly associated with SOFAS at baseline remained. This model is presented in Table 3. There were positive associations with baseline functioning for verbal memory (coefficient 0.82, 95% CI 0.33 to 1.32, *p* = 0.001), cognitive flexibility (coefficient 0.65, 95% CI 0.25 to 1.06, *p* = 0.002), and premorbid IQ (coefficient 0.12, 95% CI 0.06 to 0.18, *p* < 0.001), and negative associations for male sex (coefficient −1.87, 95% CI −3.18 to −0.57, *p* = 0.004) and depressive (coefficient −0.29, 95% CI −0.42 to −0.16, *p* < 0.001), negative (coefficient −0.48, 95% CI −0.73 to −0.24, *p* < 0.001), and positive symptoms (coefficient −0.23, 95% CI −0.39 to −0.07, *p* = 0.004).

**Table 3:** Adjusted linear mixed-effects models (*n* = 767) examining associations between neurocognitive, socio-demographic, and symptom predictor variables and (i) baseline SOFAS and (ii) baseline SOFAS and rate of change in SOFAS over time.

	(i) Baseline SOFAS (i.e., intercept)			(ii) Baseline SOFAS and rate of change over time (i.e., slope)		
	Coefficient [95% CI]	*t*	*p*	Coefficient [95% CI]	*t*	*p*
Intercept	59.63 [53.28, 65.99]	18.40	<0.001	59.05 [52.90, 65.20]	18.82	<0.001
Time	0.25 [0.10, 0.41]	3.23	0.001	NS	NS	NS
Neurocognition						
Cognitive flexibility	0.65 [0.25, 1.06]	3.14	0.002	NS	NS	NS
Verbal memory	0.82 [0.33, 1.32]	3.29	0.001	0.90 [0.42, 1.38]	3.65	<0.001
Socio-demographics						
Sex (male)	−1.87 [−3.18, −0.57]	−2.85	0.004	−3.78 [−5.22, −2.34]	−5.14	<0.001
Premorbid IQ	0.12 [0.06, 0.18]	3.97	<0.001	0.13 [0.07, 0.19]	4.53	<0.001
Clinical symptom ratings						
BPRS depressive	−0.29 [−0.42, −0.16]	−4.29	<0.001	−0.28 [−0.41, −0.15]	−4.19	<0.001
BPRS negative	−0.48 [−0.73, −0.24]	−3.91	<0.001	−0.49 [−0.74, −0.25]	−3.98	<0.001
BPRS positive	−0.23 [−0.39, −0.07]	−2.88	0.004	−0.25 [−0.41, −0.09]	−3.07	0.002
Rate of SOFAS change over time						
Time × cognitive flexibility	–	–	–	0.14 [0.06, 0.22]	3.58	<0.001
Time × gender	–	–	–	0.73 [0.49, 0.98]	5.83	<0.001

*SOFAS* Social and Occupational Functioning Assessment Scale, *Premorbid IQ* estimated premorbid intellectual functioning, *BPRS* Brief Psychiatric Rating Scale, *NS* Non-Significant

In a second model also including associations with the rate of SOFAS change over time, there were associations with baseline functioning for verbal memory (coefficient 0.90, 95% CI 0.42 to 1.38, *p* < 0.001), premorbid IQ (coefficient 0.13, 95% CI 0.07 to 0.19, *p* < 0.001), male sex (coefficient −3.78, 95% CI −5.22 to −2.34, *p* < 0.001) and depressive (coefficient −0.28, 95% CI −0.41 to −0.15, *p* < 0.001), negative (coefficient −0.49, 95% CI −0.74 to −0.25, *p* < 0.001), and positive symptoms (coefficient −0.25, 95% CI −0.41 to −0.09, *p* = 0.002). The rate of change in functioning over time varied among patients depending on their sex (male; coefficient 0.73, 95% CI 0.49 to 0.98, *p* < 0.001), indicating that males had a greater rate of improvement in functioning than females, and the baseline level of cognitive flexibility (coefficient 0.14, 95% CI 0.06 to 0.22, *p* < 0.001), indicating that patients with the lowest scores had the least improvement in functioning.

## Discussion

This study is the first to model unique associations between neurocognitive test scores and longer-term social and occupational functioning in a transdiagnostic clinical cohort of adolescents and young adults accessing youth mental health services. Of note, we observed a novel link between scores on a measure of “cognitive flexibility” (TMT-B) and the rate of improvement in social and occupational functioning over time, which was statistically independent of socio-demographics and level of symptom severity. This approach aligns with the dimensional framework endorsed by the National Institute of Mental Health’s Research Domain Criteria initiative^24^, and importantly extends diagnosis-specific links between executive functions and socio-occupational functioning^17,19,21^ to a broader transdiagnostic context. Our results expand the evidence base to suggest that cognitive flexibility may represent a meaningful and transdiagnostic target for cognitive remediation protocols in youth mental health settings.

Consistent with previous studies, we observed specific associations between baseline functioning and scores on measures of verbal memory and cognitive flexibility, clinically significant impairments of which were common and experienced by 20.6% and 23.0% of the cohort, respectively. The mechanisms underlying the associations between these domains and functioning in a transdiagnostic context are not well understood but may involve both direct (e.g., difficulty remembering instructions or inflexible decision-making) and indirect effects (e.g., mediated by social cognition or self-efficacy), as observed in schizophrenia^51,52^. Critically, cognitive flexibility also had a robust association with the rate of improvement in functioning longitudinally, such that impaired flexibility was associated with a lower rate of functional recovery over time in contact with clinical services. The independence from level of symptom severity provides clues to an enduring executive impairment linked to functioning. The measure of cognitive flexibility used in this study—the TMT-B—is thought to index higher-order skills such as the ability to flexibly switch between different task demands, in addition to other lower-order abilities such as visual search and processing speed^53^. In general populations, greater cognitive flexibility predicts a range of favorable outcomes across the life course, including better reading ability in children^54^, trait resilience to emotional events in adults^55^, and better health-related quality of life in older adults^56^. Moreover, studies in patients with mental disorders such as bipolar disorder have reported associations between impairments on the TMT-B and poorer functioning cross-sectionally and longitudinally^14,57^. Neuroimaging studies in healthy adults have revealed a distributed network of frontoparietal regions supporting cognitive flexibility^58^, a number of which are commonly altered in individuals with mental disorders. Thus, the link between cognitive flexibility and functioning observed in general populations may be amplified in individuals who have a mental disorder and neurocognitive impairment, as in our cohort wherein almost one-quarter had a clinically significant impairment in cognitive flexibility.

The link between cognitive flexibility and functional improvement may have important treatment implications. Cognitive remediation is increasingly being incorporated into treatment plans for individuals with mental disorders, with evidence that real-world functional gains are greatest when cognitive training is combined with supplemental functional skills training^59^ or other vocational interventions. Moreover, some preliminary animal modeling suggests that the adolescent brain may be better able to learn from cognitive training^60^ as a function of the unique neurobiology of adolescence (e.g., reward hypersensitivity). Further, accumulating evidence suggests that cognitive remediation in early phases of illness may yield greater than when applied in chronic phases^61,62^. Our results suggest that cognitive flexibility may represent a meaningful and transdiagnostic target for cognitive remediation, which may be enhanced when offered to young people early in the course of illness alongside other interventions targeting social and occupational functioning.

### Limitations

Several limitations need mention. First, we relied on a baseline neurocognitive assessment, which is less informative than tracking neurocognitive and functional change dynamically over time. Second, we relied on a single-item index of functioning, potentially missing specific associations with sub-domains of functioning (e.g., relating interpersonally vs. vocational performance). Third, the age range studied spans a dynamic phase of neurocognitive development. Age-related test heterogeneity may therefore have obscured age-specific effects, and our results may not be generalizable to all age groups. However, more than 80% of the sample was aged 15–25, and some research suggests that while cognitive flexibility peaks in early adulthood, it is relatively mature by later childhood^63^. Fourth, as a result of the naturalistic design of this cohort study, sample attrition over time was uncontrolled and may have biased our model estimates. For example, the number of participants retained at the 5-year follow-up timepoint was limited, and it is possible that those remaining in care for longer durations have more severe illnesses which require greater attention from clinical services. Unfortunately, we did not collect data regarding specific patterns of treatment usage (e.g., number of sessions with a psychologist). However, the naturalistic design of this study may in fact better reflect the real-world patterns of service usage and functioning. Fifth, studies in schizophrenia consistently report statistical mediation of the path from neurocognition to functional outcome by several factors which were unmeasured here, including social cognition and intrinsic motivation^51^—they are likely relevant to other major mental disorders. Finally, cognitive flexibility and set-shifting are related neurocognitive functions, but we did not observe an association between functioning and set-shifting in our final model. One possible explanation for this discrepancy is that the test used to measure cognitive flexibility (TMT-B) additionally recruits functions including visual search, processing speed, and working memory and may therefore be inherently more difficult^53^.

## Conclusions

In summary, we demonstrate for the first time a robust association between performance on a measure of cognitive flexibility and the rate of functional recovery over time in a transdiagnostic cohort of adolescents and young adults. Our results may have particular relevance for young people accessing broadly-based youth mental health services for whom impairments in cognitive flexibility may represent a treatment target for cognitive remediation in isolation or alongside functional interventions. Future studies should attempt to replicate our observations and determine the efficacy of cognitive remediation or functional interventions in individuals with impaired cognitive flexibility.

## Supplementary information

Supplementary Tables
