# Supplementary material for: Modelling associations between neurocognition and functional course in young people with emerging mental disorders: a longitudinal cohort study
Source: Transl Psychiatry. 2020 Jan 21;10:22. doi: 10.1038/s41398-020-0726-9 (PMC7026055; doi:10.1038/s41398-020-0726-9)
Supplement: Supplementary file 1 — Supplementary Tables [file 41398_2020_726_MOESM1_ESM.docx]

**Supplementary Table 1. Numbers and proportions of participants with a proforma assessment at each timepoint.**

| **Timepoint** | **N (%)** |
| --- | --- |
| Baseline | 767 (100.0) |
| Six-months | 247 (32.2) |
| One-year | 275 (35.5) |
| Two-years | 236 (30.8) |
| Three-years | 170 (22.2) |
| Four-years | 112 (14.6) |
| Five-years | 59 (7.7) |

**Supplementary Table 2. Distribution of timepoints across participants.**

| **Number of timepoints** | **N (%)** |
| --- | --- |
| One | 302 (39.4) |
| Two | 182 (23.7) |
| Three | 110 (14.3) |
| Four | 64 (8.3) |
| Five | 60 (7.8) |
| Six | 29 (3.8) |
| Seven | 20 (2.6) |

**Supplementary Table 3. Number and proportions of missing data for all predictor variables.**

| **Variable** | **Missing, N (%)** |
| --- | --- |
| Gender | 0 (0) |
| Age (baseline) | 0 (0) |
| Premorbid IQ | 57 (7.4) |
| BPRS, depressive | 85 (11.1) |
| BPRS, negative | 86 (11.2) |
| BPRS, positive | 84 (11.0) |
| BPRS, manic | 79 (10.3) |
| Processing Speed (TMT-A) | 13 (1.7) |
| Cognitive Flexibility (TMT-B) | 18 (2.3) |
| Verbal Learning (RAVLT-sum) | 28 (3.7) |
| Verbal Memory (RAVLT-A7) | 9 (1.2) |
| Sustained Attention (RVP-A) | 59 (7.7) |
| Verbal Fluency (COWAT) | 12 (1.6) |
| Working Memory (SSP) | 48 (6.3) |
| Visuospatial Learning (PAL) | 54 (7.0) |
| Set Shifting (IED) | 59 (7.7) |

**Supplementary Table 4. Mean and standard deviation of SOFAS scores at each timepoint.**

| **Timepoint** | **SOFAS (M ± SD)** | | |
| --- | --- | --- | --- |
|  | **Whole sample** | **Males** | **Females** |
| **Baseline** | 60.19 ± 10.05 | 58.24 ± 10.03 | 61.66 ± 9.63 |
| **Six-months** | 60.57 ± 9.75 | 59.89 ± 9.85 | 61.02 ± 9.95 |
| **One-year** | 60.33 ± 10.63 | 58.91 ± 11.05 | 61.62 ± 10.03 |
| **Two-years** | 61.75 ± 10.73 | 60.67 ± 11.55 | 62.16 ± 10.31 |
| **Three-years** | 61.25 ± 11.10 | 60.75 ± 12.09 | 62.57 ± 10.34 |
| **Four-years** | 61.76 ± 10.12 | 61.43 ± 9.23 | 60.44 ± 10.84 |
| **Five-years** | 58.51 ± 9.47 | 58.70 ± 11.26 | 59.92 ± 10.01 |

**Supplementary Table 5. Unadjusted linear mixed-effects models (n=767) examining associations between neurocognitive, socio-demographic, and symptom predictor variables and (i) baseline SOFAS and (ii) rate of SOFAS change.**

| **(i) SOFAS intercept (i.e. baseline)** | | | |  | **(ii) SOFAS slope (i.e. change over time)** | | | |
| --- | --- | --- | --- | --- | --- | --- | --- | --- |
| **Predictors** | **Coefficient [95% CI]** | **t** | **p** |  | **Predictors** | **Coefficient [95% CI]** | **t** | **p** |
| Sex (male) | -2.65 [-3.98, -1.31] | -3.89 | <.001 |  | Time x Gender (male) | 0.39 [0.16, 0.61] | 3.39 | <.001 |
| Age | 0.02 [-0.15, 0.18] | 0.20 | .845 |  | Time x Age | 0.03 [0.01, 0.05] | 3.13 | .002 |
| Premorbid IQ | 0.18 [0.12, 0.24] | 5.96 | <.001 |  | Time x Premorbid IQ | 0.00 [0.00, 0.00] | 4.04 | <.001 |
| BPRS Depressive | -0.33 [-0.45, -0.20] | -4.93 | <.001 |  | Time x BPRS Depressive | 0.01 [0.00, 0.02] | 2.33 | .020 |
| BPRS Negative | -0.89 [-1.13, -0.66] | -7.39 | <.001 |  | Time x BPRS Negative | 0.02 [-0.00, 0.04] | 1.90 | .057 |
| BPRS Positive | -0.49 [-0.65, -0.32] | -5.90 | <.001 |  | Time x BPRS Positive | 0.01 [0.00, 0.03] | 2.14 | .033 |
| BPRS Mania | -0.21 [-0.40, -0.02] | -2.15 | .032 |  | Time x BPRS Mania | 0.02 [0.01, 0.03] | 2.67 | .008 |
| Processing Speed | 1.78 [1.20, 2.35] | 6.02 | <.001 |  | Time x Processing Speed | 0.15 [0.03, 0.28] | 2.48 | .013 |
| Cognitive Flexibility | 1.30 [0.91, 1.69] | 6.51 | <.001 |  | Time x Cognitive Flexibility | 0.15 [0.07, 0.23] | 3.79 | <.001 |
| Verbal Learning | 1.56 [1.08, 2.04] | 6.33 | <.001 |  | Time x Verbal Learning | 0.13 [0.02, 0.24] | 2.41 | .016 |
| Sustained Attention | 1.15 [0.77, 1.53] | 5.89 | <.001 |  | Time x Sustained Attention | 0.03 [-0.05, 0.10] | 0.73 | .468 |
| Verbal Memory | 1.63 [1.15, 2.11] | 6.62 | <.001 |  | Time x Verbal Memory | 0.13 [0.02, 0.24] | 2.42 | .016 |
| Verbal Fluency | 1.43 [0.85, 2.02] | 4.81 | <.001 |  | Time x Verbal Fluency | 0.03 [-0.08, 0.15] | 0.56 | .574 |
| Working Memory | 1.48 [0.93, 2.03] | 5.25 | <.001 |  | Time x Working Memory | 0.15 [0.04, 0.25] | 2.65 | .008 |
| Visuospatial Learning | 0.87 [0.49, 1.25] | 4.47 | <.001 |  | Time x Visuospatial Learning | 0.01 [-0.08, 0.09] | 0.12 | .904 |
| Set Shifting | 0.53 [0.17, 0.90] | 2.88 | .004 |  | Time x Set Shifting | 0.06 [-0.02, 0.13] | 1.42 | .156 |

**Note**: ‘SOFAS’ = Social and Occupational Functioning Assessment Scale; ‘Premorbid IQ’ = Estimated premorbid intellectual functioning; ‘BPRS’ = Brief Psychiatric Rating Scale.
